# Drought stress increases total C released from roots

**DOI:** 10.1093/aob/mcag007

**Published:** 2026-01-08

**Authors:** Danielle E M Ulrich, Kelsey Flathers, Hannah M Goemann, Rebecca C Mueller, Brent M Peyton

**Affiliations:** Department of Ecology, Montana State University, Bozeman, MT 59717, USA; Ecostudies Institute, Olympia, WA 98507, USA; Department of Plant Pathology, University of California Davis, Davis, CA 59616, USA; United States Department of Agriculture, Agricultural Research Service, Western Regional Research Center, Albany, CA 94710, USA; Department of Chemical and Biological Engineering and Center for Biofilm Engineering, Montana State University, Bozeman, MT 59717, USA

**Keywords:** Root exudates, rhizodeposition, drought stress, C allocation, metabolomics, plant–microbe interactions, roots

## Abstract

**Background and Aims:**

Root exudation and rhizodeposition are critical pathways for plant carbon (C) allocation to soil, influencing soil organic C stability and ecosystem functioning under changing climates. However, the effects of drought stress on these below-ground processes remain poorly understood. We provide an updated and comprehensive assessment of the current state of knowledge on how drought stress affects root-derived C fluxes (root exudates, rhizodeposition) across diverse functional types and experimental setups to direct future research.

**Methods:**

We conducted a meta-analysis to quantify how drought stress affects total C and other compound classes in root exudation and rhizodeposition.

**Key Results:**

Our analysis included 40 data points spanning diverse functional types, ecosystems, sampling methods and experimental conditions. We observed that root-released total C significantly increased in response to drought stress. Among compound classes, carbohydrates and organic acids also increased in response to drought stress, suggesting these compound classes may underlie total C responses in root exudation and rhizodeposition. The variables that explained the most variance in total C response ratio were ecosystem, flowering type, cotyledon type, functional type and drought intensity.

**Conclusions:**

To direct future research, our analysis identified knowledge gaps, particularly the need for studies to examine trees and shrubs, field-based research, broader drought intensity ranges, and measurements of total C, compound classes and specific compounds when possible. Improving our understanding of root-derived C responses to stress is crucial for predicting terrestrial C cycling dynamics and ecosystem function under increased drought frequency and intensity.

## INTRODUCTION

The world’s soils store more carbon (C) in the form of soil organic C (SOC) than is stored in the atmosphere and all living vegetation combined (Lefèvre *et al.*, 2017). SOC stability is tightly linked to the functioning and productivity of natural and agricultural ecosystems (Amundson *et al.*, 2015; Crowther *et al.*, 2019). However, the uncertain consequences of increasing drought stress on SOC challenge our ability to predict terrestrial C cycling dynamics under future climates. A key mechanism involved in SOC dynamics is rhizodeposition, the release of plant-derived organic C into the soil. Rhizodeposition is defined as all material lost from roots, including root exudates, dead root cells and materials, volatile organic compounds (VOCs) and gases such as CO_2_ and ethylene (Nguyen, 2003; Jones *et al.*, 2004; Pausch and Kuzyakov, 2018). Root exudates are soluble organic compounds ranging from low-molecular weight compounds (e.g. sugars, organic acids, amino acids, phenolics) to high-molecular weight compounds (e.g. some enzymes) (Bais *et al.*, 2006; Badri and Vivanco, 2009). Interest in root exudate and rhizodeposition research has grown in recent years due to potential applications in agriculture and crop productivity (Busby *et al.*, 2017; Zambelli *et al.*, 2025), engineering the rhizosphere for soil C sequestration (Wang and Kuzyakov, 2024), disease management (Lamichhane *et al.*, 2024) and ecosystem function (Villarino *et al.*, 2021; Ma *et al.*, 2022; Panchal *et al.*, 2022; Delory *et al.*, 2024).

The effects of root-derived compounds on ecosystem function occur through multiple pathways (Mendes *et al.*, 2013; Brunn *et al.*, 2025) including: chemical signalling between plants and other organisms (Bais *et al.*, 2006; Baetz and Martinoia, 2014; Haichar *et al.*, 2014; Hassan *et al.*, 2019); maintaining rhizosphere water content (Carminati *et al.*, 2016; Ulrich *et al.*, 2019); forming mineral-associated SOC (Sokol *et al.*, 2019); destabilizing SOC through disruption of mineral organic associations (Keiluweit *et al.*, 2015; Sokol *et al.*, 2019; Chari and Taylor, 2022); priming microbial community activity (Kuzyakov and Domanski, 2000; Dijkstra *et al.*, 2021; Yan *et al.*, 2023); increasing nutrient availability to plants and microbes (Chai and Schachtman, 2022); inhibiting growth of soil-borne pathogens (Lanoue *et al.*, 2010); shaping microbial communities (van Dam and Bouwmeester, 2016; Goemann *et al.*, 2024); and supporting microbiota to facilitate adaptation to stress (Baetz and Martinoia, 2014; Rolfe *et al.*, 2019). Considering these functional roles, root exudation and rhizodeposition are central to SOC stability and long-term C sequestration, and underlie ecosystem and agricultural productivity responses to environmental change (de Vries *et al.*, 2020; Williams and de Vries, 2020; Jha *et al.*, 2024; Kruthika *et al.*, 2024).

Drought stress can reduce a plant’s ability to assimilate C through photosynthesis and affect how C is allocated to essential functions, including above- and below-ground growth, reproduction, metabolism and stress resistance (Hartmann and Trumbore, 2016). Among these C sinks is the allocation of C directly to soil through rhizodeposition and root exudates. However, how drought stress affects these below-ground processes remains poorly understood, largely due to the challenges of quantifying root exudation and rhizodeposition (Oburger and Jones, 2018; Vives-Peris *et al.*, 2020; Kruthika *et al.*, 2024; Brunn *et al.*, 2025). First, the quantity and composition of exudation and rhizodeposition are highly variable temporally and spatially and affected by multiple factors. For example, the quantity and composition of root exudates can vary with biotic and abiotic stress type, duration and severity (Williams and de Vries, 2020), over the life cycle of a plant (Lynch and Whipps, 1990; Nguyen, 2003; Zhalnina *et al.*, 2018), in different root types and regions (Tiziani *et al.*, 2022; Gao *et al.*, 2024; Tixier *et al.*, 2025), diurnally (McLaughlin *et al.*, 2023) and seasonally (Gao *et al.*, 2024), and by plant species (Kuzyakov and Domanski, 2000), functional type and environmental conditions (Williams *et al.*, 2022), including drought severity (Ulrich *et al.*, 2022) and recovery (Gargallo-Garriga *et al.*, 2018). Second, numerous methods are used to quantify root exudation and rhizodeposition across studies, with each method having distinct advantages and disadvantages, and few studies comparing methods in the same experiment (Oburger and Jones, 2018; Williams *et al.*, 2021). Common methods include hydroponic systems, leachate collection, exudation traps, *in situ* collection with rhizotrons and isotope labelling. Additionally, it is extremely difficult at the experimental level to accurately distinguish root exudates and rhizodeposits in space and time (Jones *et al.*, 2009). Finally, the mechanism of root exudation, including whether it is an active or passive process, remains poorly understood (Farrar and Jones, 2000; Prescott *et al.*, 2020).

Given these factors, estimates of the amount and rate of C allocated to root exudation and rhizodeposition vary widely. Current estimates are that ∼16–50 % of photosynthetically fixed C is allocated below ground (Kuzyakov and Domanski, 2000; Derrien *et al.*, 2004), with roughly 50 % of that going to root biomass, 33 % to rhizosphere respiration, and anywhere from 17 to 90 % to rhizodeposition and root exudation (Lynch and Whipps, 1990; Jones *et al.*, 2004, 2009; Haichar *et al.*, 2014, 2016; Chari *et al.*, 2024). Overall, 2–40 % of photosynthetically fixed C may be released via rhizodeposition and root exudation (Lynch and Whipps, 1990; Nguyen, 2003; Jones *et al.*, 2004, 2009; Pinton *et al.*, 2007; Badri and Vivanco, 2009; Pausch and Kuzyakov, 2018; Sasse *et al.*, 2018; Jha *et al.*, 2024). These uncertainties in quantification in isolated studies limit our ability to understand how environmental stresses such as drought impact C allocation to root exudation and rhizodeposition and consequently our ability to understand and predict terrestrial C storage and cycling. Meta-analysis can improve our understanding of the broad-scale effects of drought stress on root exudation and rhizodeposition, especially in summarizing patterns and identifying knowledge gaps (Gurevitch *et al.*, 2018).

A previous review (Preece and Peñuelas, 2016) analysed nine studies published between 1990 and 2015 and found that rhizodeposition increased under moderate drought conditions but was variable under more severe drought conditions. The authors noted that the scarcity of well-defined studies precluded a complete meta-analysis. We built on this study by synthesizing the growing body of literature that included more studies, functional types and collection methods. Our meta-analysis sought to systematically survey current literature to (1) quantify how drought stress affects the direction and magnitude of changes in root exudate and rhizodeposition quantity and composition, (2) identify how root exudate and rhizodeposition responses to drought differ by functional type, developmental stage, experimental conditions and sampling method, and (3) identify current knowledge gaps to direct future research.

## MATERIALS AND METHODS

### Study selection

We searched ISI Web of Science for peer-reviewed studies from 1980 to 2023 using the following search terms: ‘root exudat*drought’. In addition, we manually added publications not found in our initial Web of Science search that were included in previous reviews on the topic (Preece and Peñuelas, 2016; Chai and Schachtman, 2022). This yielded a total of 250 studies with 216 studies that referenced drought stress. We screened publications for studies that included: (1) a definable manipulation of water stress level as the treatment; (2) a comparison between treatment and control groups; (3) measurements of mean, error and sample size of root exudate or rhizodeposition quantity (concentration, rate), compound classes and/or specific compounds, and (4) root dry biomass measurements for control and treatment groups to standardize all measurements. We eliminated reviews, commentaries and publications that used polyethylene glycol (PEG) to induce osmotic stress in a solution, and publications that reported only root exudate relative composition and not quantity. Our screening process yielded 29 studies on drought stress that we included in subsequent analyses (references in Supplementary Data Literature Cited).

### Data acquisition

For each study, we surveyed the response variable (root C type; root exudate, rhizodeposition), response variable quantity (concentration or rate), compound type collected (total C, total N, protein and amino acids, carbohydrates and related compounds, organic acids, and lipids and related compounds (fatty acids, fatty alcohols, sterols)), ecosystem (agricultural, grassland, shrubland, forest), life history (annual, perennial), flowering type (angiosperm, gymnosperm), cotyledon type (monocot, dicot), functional type (grass, forb, legume, grass/forb/legume mix, broadleaf tree, conifer, shrub), developmental stage (seedling, juvenile, adult), species cultivated for agriculture or not (cultivated, wild), experimental setup (greenhouse, field), and sampling method (exudation trap, soil–hydroponic hybrid, isotope-based, leachate collection, soil suspension). We define the exudation trap method as using an exudation trap (e.g. cuvette) to collect exudates from intact roots still attached to a live plant, the soil–hydroponic hybrid method as transplanting the entire intact plant and root system to a hydroponic system and/or C-free medium for exudate collection, the isotope-based method as using isotope tracers and labelling, the leachate collection method as soaking roots in a solution and collecting exudates from the solution without excavation, and the soil suspension method as rinsing roots and rhizosphere soil with a buffer and collecting the filtered suspension.

Mean root exudate and rhizodeposition values of total C and compound classes, samples sizes and root dry weight for treatment and control groups were collected from each study. We used ImageJ (Schneider *et al.*, 2012) to digitize figures and determine mean and standard error values if raw values were not provided. Some publications measured multiple drought treatments (e.g. severity), species, genotypes of a single species, species/genotypes grown together and grown separately, compound classes and/or specific compounds. In these instances, the response of each drought treatment, species, genotype or compound class was considered its own data point, resulting in 40 data points measuring a form of total C and 39 data points measuring other compound classes (total C, total N, protein and amino acids, carbohydrates, lipids and related compounds, organic acids). When specific compounds were measured, we averaged the effect sizes of individual compounds for the respective compound class (i.e. one effect size per compound class). We focused on compound classes rather than specific compounds because the few studies that analysed specific compounds did not always measure the same specific compound. Total C (also known as specific exudation rate) was calculated per gram of root biomass and included: total organic carbon (TOC), dissolved organic carbon (DOC), soluble C, extractable organic carbon (EOC) in soil, percentage atom excess of ^13^C and percentage atom excess of ^14^C and plant-derived SOC. Drought intensity was calculated based on Preece and Peñuelas (2016), in which the duration of reduced water (in days) is multiplied by the reduction in water relative to the control (as a proportion).

### Statistical analysis

All statistical analysis was conducted in R version 2025.05.1 + 513 and MetaWin3 software version 3.1.0 (Rosenberg, 2024). We calculated the effect of drought treatment (on total C and compound classes released as exudates or rhizodeposits) as the response ratio (RR). The RR was calculated as the natural log of the difference in mean treatment (drought) value ($\bar{X}$_E_) and the mean control (ambient) value ($\bar{X}$_c_) (Hedges *et al.*, 1999). The natural logarithm of the response ratio (1) linearizes the metric, so that the RR is affected equally by changes in the numerator and denominator, and (2) normally distributes RR. An RR of 0 indicates no effect of drought treatment on the response variable. If a 95 % confidence interval did not intersect with 0, the RR was considered significant. The RR was determined for total C, compound classes and specific compounds. If a study included specific compounds, we averaged the RR of each specific compound to determine the mean RR for each compound class so that each study had one mean RR for each compound class. Total C RR was determined for the following: ecosystem (agricultural, grassland, shrubland, forest); life history (annual, perennial); flowering type (angiosperm, gymnosperm); cotyledon type (monocot, dicot); functional type (grass, forb, legume, grass/forb/legume mix, broadleaf tree, conifer, shrub); developmental stage (seedling, juvenile, adult); species cultivated for agriculture or not (cultivated, wild); experimental setup (greenhouse, field); type of root C (root exudate, rhizodeposition); and sampling method (exudation trap, soil-hydroponic-hybrid, isotope-based, leachate collection, soil suspension).

For the 40 total C RR data points, we used two different approaches to test for the presence of publication bias: Egger’s regression test (Egger *et al.*, 1997) and a rank correlation test based on Kendall’s *τ* (Kendall, 1938; Begg and Mazumdar, 1994). We used a random effects model, which accounts for both within- and between-study variation with 95 % confidence intervals and bootstrapped confidence intervals with 999 iterations to quantify the level of heterogeneity (*Q* = total heterogeneity; *I*^2^ = percentage of total variation that is due to heterogeneity rather than chance (sampling error) among studies).

Several studies contributed multiple RR data points (due to multiple species or treatments within a study), violating the independence assumption of a standard meta-analysis (Hedges *et al.*, 2010). To address this, we adjusted the sampling variance of each RR by the number of RR data points contributed by that study. This approach ensures that each study contributes approximately equal weight to the analysis.

To identify factors that might explain the heterogeneity in total C RR and to examine how total C RR varied within different categories, we used random effects categorical models of total C for the categories (variables) listed above (ecosystem, life history, flowering type, cotyledon type, functional type, developmental stage, species cultivated for agriculture or not (wild), experimental setup, root C type, sampling method). This effort resulted in *Q*_total_, which is the total heterogeneity among RRs and equals the sum of *Q*_within_ and *Q*_between_ (*Q*_total_ = *Q*_within_ + *Q*_between_). *Q*_within_ is the heterogeneity within subgroups of a category (e.g. forest, grassland, agriculture and shrubland within the category of ecosystem). *Q*_between_ is the variance explained by the variable (e.g. ecosystem) between subgroups. *P*-values <0.05 for *Q*_between_ were used to indicate that subgroups within a category (e.g. ecosystem) differed significantly and that the variable (e.g. ecosystem) is statistically important in explaining variance. *τ*^2^ is the estimate of pooled variance across all studies, representing how much true RRs vary. Residual *τ*^2^ is the variance that remains within subgroups after grouping by each variable (i.e. a low residual *τ*^2^ indicates that that variable explains nearly all of the variance or that points within subgroups are homogeneous). The variables that explained the most variance (and had *P* < 0.05) had the smallest residual *τ*^2^ and largest percentage reduction in *τ*^2^.

To evaluate how total C varied with drought intensity, we used a random effects continuous model in an approach similar to that described above. To determine if total C RR was significantly related to drought intensity, we used a linear mixed effects model fitted by restricted maximum likelihood (REML) with total C RR as the response variable, drought intensity as a fixed effect, and study as a random effect to account for studies that contributed multiple data points due to multiple drought treatments and/or species within a single study. Outliers were checked using the interquartile range (IQR) method (data points beyond quartile 1 ± 1.5 × IQR) and influence diagnostics (Cook’s distance >4/*n*), and comparing models with and without the outlier to assess the impact of outlier removal on regression parameters.

## RESULTS

### Summary statistics

**Fig. 1.**
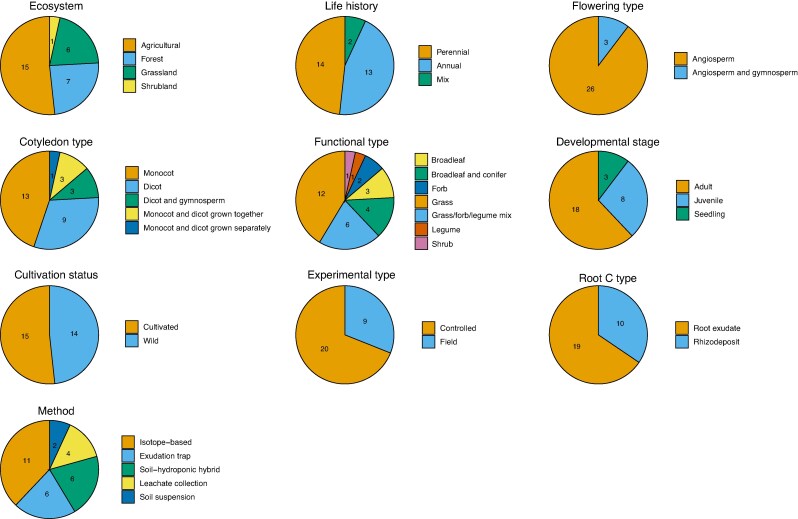
Number of drought stress studies in the analysis in the following categories: ecosystem (forest, grassland, agricultural, shrubland); life history (perennial, annual); flowering type (gymnosperm, angiosperm); cotyledon type (monocot, dicot); functional type (grass, forb, legume, grass/forb/legume mix, broadleaf tree, conifer, shrub); developmental stage (seedling, juvenile, adult); cultivation status (wild, cultivated); experimental type (controlled: greenhouse/growth chamber, field); root C type (root exudate, rhizodeposition); and method (exudation trap, soil–hydroponic hybrid, isotope-based, leachate collection, soil suspension).

We identified 29 studies that matched our selection criteria (references in Supplementary Data Table S1 and Supplementary Data Literature Cited). The 29 studies varied in species, ecosystem, functional type, experimental setup and sampling methods (Fig. 1, Supplementary Data Table S1). Overall, the majority of studies investigated agricultural ecosystems (an ecosystem primarily managed to produce food, fuel or fibre; 15 studies), perennials (14 studies), angiosperms (26 studies), monocots (13 studies), grass species (12 studies), the adult developmental stage (18 studies), cultivated species (species grown as either crops or forage; 15 studies), controlled experimental settings (greenhouse or growth chamber; 20 studies), root exudates (19 studies) and the isotope-based sampling method (11 studies). Root exudation and rhizodeposition differed in the number of sampling methods used. Root exudation was measured using five methods: isotope-based (two studies), exudation trap (six studies), soil–hydroponic hybrid (six studies), leachate collection (four studies) and soil suspension (one study). In contrast, rhizodeposition was measured with only the isotope-based (nine studies) and soil suspension methods (one study). We observed that the majority of data points measured total C (*n* = 40 data points) in all functional types over a broad drought intensity range (1–147) while fewer data points measured the other compound classes (total *N*, *n* = 5; protein and amino acids, *n* = 10; carbohydrates, *n* = 6; organic acids, *n* = 9; lipids and related compounds, *n* = 9) over a drought intensity range (1–144) that was skewed towards lower values (1–45, except 2 organic acid data points at an intensity of 144).

### Drought effects on root-released total C

**Fig. 2.**
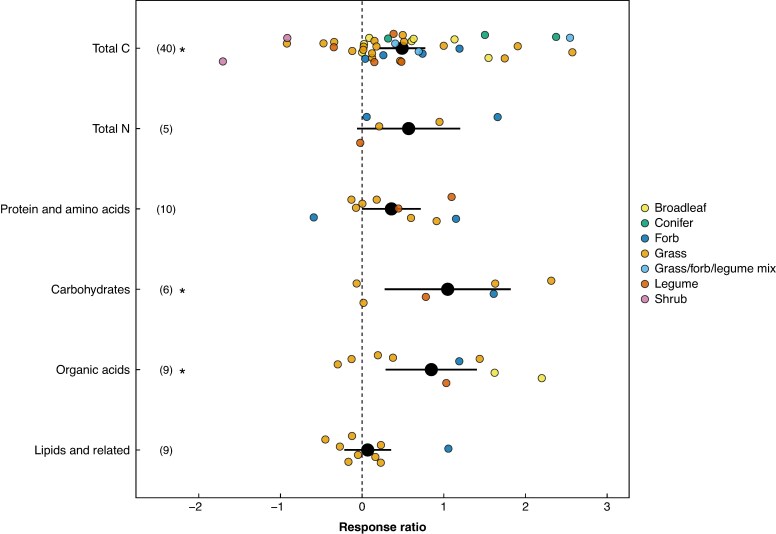
Response ratio describing the effect of drought on root exudate compound classes (total compound released per root biomass). Values are organized by compound class: total C; total N; protein and amino acids; carbohydrates; organic acids; and lipids and related compounds (fatty acids, fatty alcohols, sterols). Error bars represent 95 % confidence intervals. Error bars that do not overlap with a response ratio RR of 0 indicate a significant effect of drought on root exudate compound class (*). Numbers in parentheses indicate the number of data points in each compound class.

Total C significantly increased under drought stress with a mean RR of 0.41 (95 % CI 0.23, 0.59; bootstrapped CI 0.19, 0.69) (Fig. 2). The back-transformed RR of 1.51 indicates that drought treatment increased total C released by 51 % over the control. We found no evidence of publication bias based on the Egger’s regression test (*P* = 0.89) and the rank correlation test (*τ* = −0.0985, *P* = 0.38). We detected moderate heterogeneity across studies (*Q* = 62.37, d.f. = 38, *P* = 0.0076, *I*^2^=39.07 %, 95 % CI 10.26 %, 58.63 %), justifying using a random effects model. *τ*^2^ was 0.1681, which is the estimate of pooled variance across all studies, representing how much true effect sizes vary between studies (between-study variance). The variables that explained the most variance and resulted in the largest reduction in *τ*^2^ were ecosystem (*P* < 0.001, 51.99 % reduction), flowering type (*P* < 0.001, 53.30 %), cotyledon type (*P* = 0.0001, 42.30 %) and functional type (*P* = 0.0001, 17.19 %) (Supplementary Data Table S2).

**Fig. 3.**
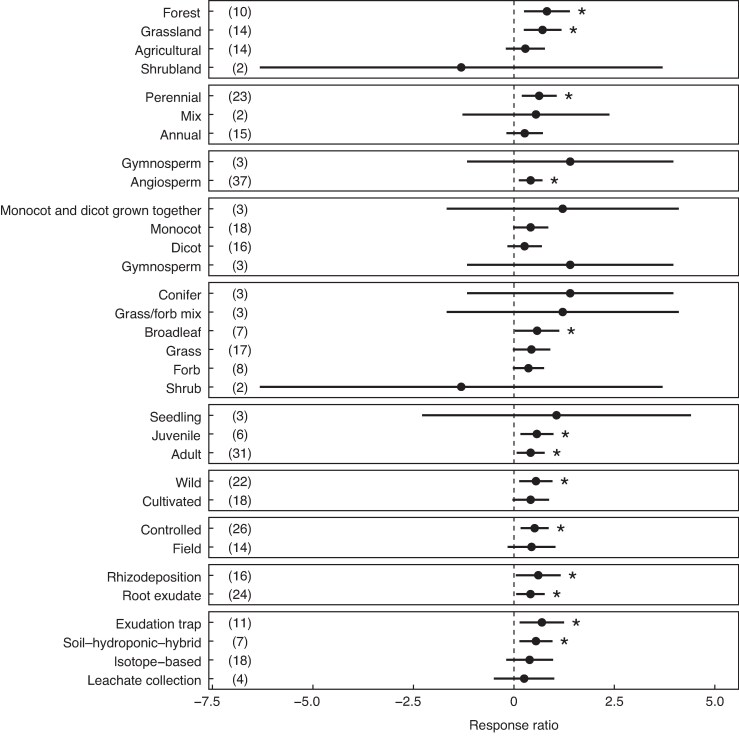
Response ratio describing the effect of drought on total C released per root biomass for the following categories: ecosystem (agricultural, grassland, shrubland, forest); life history (annual, perennial); flowering type (angiosperm, gymnosperm); cotyledon type (monocot, dicot); functional type (grass, forb, legume, grass/forb/legume mix, broadleaf tree, conifer, shrub); developmental stage (seedling, juvenile, adult); species cultivated for agriculture or not (cultivated, wild); experimental setup (greenhouse, field); root C type (rhizodeposition, root exudate); and sampling method (exudation trap, soil–hydroponic hybrid, isotope-based, leachate collection, soil suspension). Note that no studies that measured total C used the soil suspension method, so it is not listed in the figure. Error bars represent 95 % confidence intervals. Error bars that do not overlap with a response ratio of 0 indicate a significant effect of drought on total C (*). Numbers in parentheses indicate the number of data points in each category level. Total C differed significantly between groups within each of the following categories: ecosystem; flowering type; cotyledon type; and functional type (Supplementary Data Table S2). Supplementary Data Table S2 presents the performance of each category (variable) in explaining heterogeneity.

Carbohydrates and organic acids also significantly increased under drought stress (RR = 1.05, 95 % CI 0.04, 2.06 and RR = 0.847, 95 % CI 0.289, 1.41, respectively; Fig. 2). All other compound classes (total N, protein, lipids) showed a positive mean response to drought, albeit not significant. Total C data points included all plant functional types (broadleaf tree, conifer, forb, grass, grass/forb/legume mix, legume, shrub) while the other compound classes were only measured on grass, forb, legume and broadleaf tree types. When examining the effect of other functional classes, developmental stage, experimental setup and method on drought RR of total C, we observed that the RR of total C was also significantly positive in the following categories: forest and grassland ecosystems; perennials; angiosperms; broadleaf tree types; juveniles and adults; wild (non-cultivated) species; controlled experimental conditions (e.g. greenhouse, growth chamber); rhizodeposition and root exudates; and the exudation trap and soil–hydroponic hybrid methods (Fig. 3). Total C RR differed significantly between subgroups within ecosystem, flowering type, cotyledon type, functional type, cultivation status and sampling method (Supplementary Data Table S2).

**Fig. 4.**
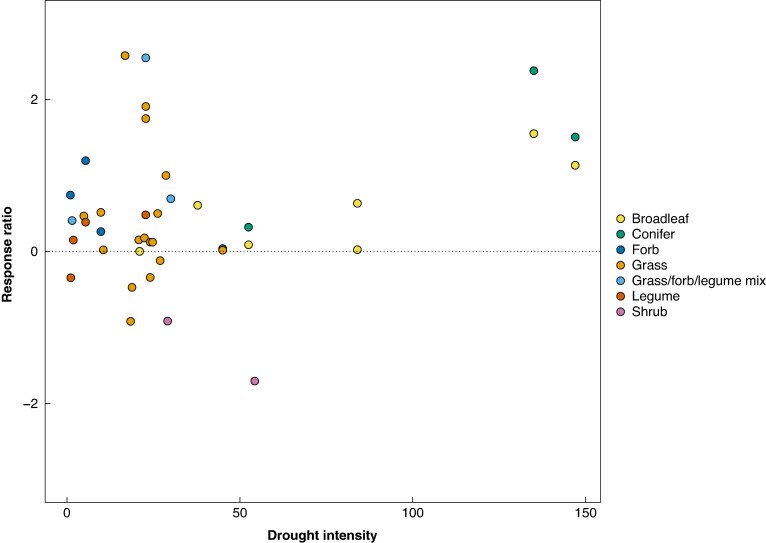
The response ratio of total C did not significantly change with drought intensity based on a linear mixed effects model fit by restricted maximum likelihood (REML) with total C response ratio as the response variable, drought intensity as a fixed effect, and study as a random effect (*P* = 0.174). Colours indicate functional type (grass, forb, legume, grass/forb/legume mix, broadleaf tree, conifer, shrub).

Drought intensity reduced *τ*² by 40.33 %, indicating its importance in explaining the variance of total C RR (*P* < 0.001; Supplementary Data Table S2). However, total C RR did not significantly change with increasing drought intensity after accounting for study (*P* = 0.174, Fig. 4), albeit spanning a drought intensity range (1–147), all seven functional types and all studies.

## DISCUSSION

### Drought increases total C released from roots

Our analysis quantified how root exudation and rhizodeposition responded to drought stress and drought intensity by surveying and analysing the growing body of literature. In general, root exudation and rhizodeposition of total C per gram of root biomass increased in response to drought. Albeit varying in significance, it was remarkable to observe that the vast majority of mean total C RR for different compound classes and categories (ecosystem, life history, flowering type, cotyledon type, functional type, developmental stage, methods, etc.) was positive. This result is consistent with Preece and Peñuelas (2016), who also found that rhizodeposition increased under moderate drought conditions. One study, for example, observed that after 5 years of severe drought during the growing season, mature *Picea abies* and *Fagus sylvatica* trees maintained levels of root exudation similar to those of control trees by increasing the proportion of C allocated to root exudation (Brunn *et al.*, 2022). Despite drought-induced reductions in above-ground C assimilation and growth, plants may allocate C to below-ground processes (Karst *et al.*, 2017; Liese *et al.*, 2018; Preece *et al.*, 2018; Brunn *et al.*, 2022; Ulrich *et al.*, 2022). The mechanism of C allocation to root exudates remains unclear, having been hypothesized both as a process that reflects the plant’s ‘needs’, controlled by factors drawing C into the rhizosphere, such as root colonization by fungi, as well as a process that reflects the resources the plant has ‘in surplus’, where the more C that is allocated to roots, the more C that ought to be exuded (Karst *et al.*, 2017; Prescott *et al.*, 2020). Others have proposed that root exudation may be more of an active process (Canarini *et al.*, 2019). Regardless of the exact mechanism, plants may respond to drought by reducing metabolism in shoots and concurrently upregulating root metabolic activity, and potentially increasing carbohydrate accumulation in roots, to buffer the negative impacts of reduced soil water availability (Gargallo-Garriga *et al.*, 2014). Plants commonly increase biomass allocation to roots in response to drought to forage for water (e.g. Meier and Leuschner, 2008; Ramírez-Valiente *et al.*, 2019; Guasconi *et al.*, 2023). The addition of root exudates to soil can increase soil moisture-holding capacity and change the physical soil structure to improve root growth through compacted soils (Roberson and Firestone, 1992; Oleghe *et al.*, 2017; Ulrich *et al.*, 2019). Given microbial substrate preference (Zhalnina *et al.*, 2018), the addition of root exudate compounds also can enhance the growth of microbes that support plant drought resistance (Worchel *et al.*, 2013; Sasse *et al.*, 2018; Shade and Stopnisek, 2019). For example, certain root exudate compounds can initiate microbial activity (through rhizosphere priming) (Zhu *et al.*, 2014; Wang *et al.*, 2016), and microbes can stimulate stress signalling (e.g. sugars, amino acids) and the production of phytohormones to increase root growth for water uptake (Calvo *et al.*, 2017).

### Total C released from roots did not increase with drought intensity

Drought intensity was the fourth most prominent variable explaining the variation among data points (after ecosystem, flowering type and cotyledon type). However, total C in root exudation and rhizodeposition did not significantly change with increasing drought intensity. This may have occurred due to the paucity of data points (only broadleaf and conifer tree species), especially at higher drought intensities. The lack of studies on grasses, forbs and shrubs at similarly high drought intensities also may contribute to the more variable rhizodeposition responses at higher drought intensities observed by Preece and Peñuelas (2016). Trees are larger, longer-lived functional types than grasses, forbs and shrubs so they can be exposed to higher cumulative drought intensities, a result of drought severity and duration, as well as have more time to develop a rhizosphere microbial community. Although most studies measured root exudation and rhizodeposition during a single growing season, this does not fully capture root C dynamics that occur over longer time periods, which is important to consider for understanding broad-scale drivers of terrestrial C cycling. One study on mature trees in a Mediterranean forest found that root exudation rates varied significantly over the course of the year, with lower rates in the wet season and a general increase in exudation rates during the dry season in late summer and early fall (autumn), when soil water and nutrients are less available (Jakoby *et al.*, 2020). Additionally, Liese *et al.* (2018) saw a significant increase in root exudation rates in temperate tree species in the early growing season and a marginal increase in the late summer season after 2 years of experimental summer drought. In addition to quantifying seasonal patterns of exudation (Gao *et al.*, 2024), diurnal fluctuations in root exudation also need to be considered (McLaughlin *et al.*, 2023).

### Compound classes differed in significance of positive drought response

Although all compound classes showed a positive response to drought stress, only carbohydrates and organic acids (and total C) exhibited a significant positive response to drought (i.e. whether confidence intervals intersected 0 or not), suggesting that these classes strongly contributed to the significant drought-induced increase in total C released from roots. Carbohydrates in the form of sugars (e.g. sucrose, fructose, glucose, pinitol, myo-inositol) either increased in concentration or were only detected within plants under drought conditions (Canarini *et al.*, 2016; Calvo *et al.*, 2019). Since drought-induced declines in growth can result in surplus C (Prescott *et al.*, 2020), higher root exudation rates may be a result of higher C supply in root tissues (Farrar *et al.*, 2003). Levels of root non-structural carbohydrates (NSCs; sucrose, glucose, fructose, starch) were positively related to root exudation rates under drought in *Populus tremuloides* seedlings (Karst *et al.*, 2017). NSCs serve as substrates for growth, energy, stress signalling and osmoregulatory solutes used to retain water, maintain turgor and sustain cellular function during drought (Dietze *et al.*, 2014). However, the direction and magnitude of NSC shifts in response to drought in above- and below-ground tissues vary by species and experimental setup, and only one study to our knowledge measured both root NSCs and root exudation (Karst *et al.*, 2017). Future research should measure both NSCs in plant tissues and root exudate composition in response to drought to better understand the effects of drought on plant C allocation and the relationships between plant C sinks (plant tissues, root exudates).

In addition to carbohydrates, we observed that organic acids increased in response to drought. Organic acids may increase the availability of soil nutrients for plants and microbes because organic acids can liberate organic matter from minerals and increase organic matter availability to microbes (Keiluweit *et al.*, 2015). Increased soil nutrient availability may be beneficial for drought-stressed plants and microbes (Gong *et al.*, 2005; Hueso *et al.*, 2011). Root exudate composition can shape the taxonomic and functional composition of the rhizosphere microbial community by supporting or suppressing the growth and activity of certain microbes, given microbial substrate preference (Zhalnina *et al.*, 2018; Canarini *et al.*, 2019), which in turn may improve plant response to drought. The release of different compounds into the rhizosphere may influence the core rhizosphere microbiome (McLaughlin *et al.*, 2023) and induce niche differentiation, competitive exclusion and cross-feeding between soil microbes (Jacoby and Kopriva, 2019), important factors to consider for rhizosphere microbiome engineering for agricultural applications (Busby *et al.*, 2017). Therefore, examining specific C classes in addition to total C is critical for improving our understanding of plant–microbe interactions.

The five studies that measured specific compound classes in root exudates showed that plants shifted root exudation composition in response to drought stress, but the direction varied by specific compounds in each class (e.g. Gargallo-Garriga *et al.*, 2018; Ulrich *et al.*, 2022). For example, although the compound class of proteins and amino acids did not increase in response to drought, individual amino acids such as proline increased significantly in the root exudates of *Bouteloua gracilis*, *Glycine max*, *Helianthus annuus* and *Quercus ilex* (Canarini *et al.*, 2016; Gargallo-Garriga *et al.*, 2018; Ulrich *et al.*, 2022). Proline was hypothesized to be closely associated with drought protection and osmotic adjustment to maintain cell turgor (Good and Zaplachinski, 1994) and with the production of mucilage to improve root–soil hydraulic conductivity and increase soil moisture-holding capacity (Roberson and Firestone, 1992; Carminati *et al.*, 2016). Quantifying general compound classes as well as targeting specific compounds (e.g. those that are commonly associated with drought response, such as proline, and/or those that may drive root exudation, such as carbohydrates or organic acids) may be most useful for understanding root C responses to drought. However, in contrast to total C, which was measured over a drought intensity range of 1–147, specific compound classes were measured over a relatively narrow drought intensity range (1–45, with only two organic acid data points measured at an intensity of 144) via shorter drought periods or mild drought conditions, and fewer functional types and studies, indicating the need for future research to measure root exudate composition including compound classes and specific compounds at higher drought intensities to more fully understand root exudate response to drought.

### Drought response differed between functional types and ecosystems

The majority of functional types and ecosystems had positive mean total C RR. However, the significance (i.e. whether confidence intervals intersected 0 or not) of the effect of drought on total C released to root exudates and rhizodeposition differed between plant functional types and ecosystems. This is consistent with previous work that has observed root exudation differences between species, ecotypes, accessions and ecosystems (Micallef *et al.*, 2009; Brunn *et al.*, 2025). Our expanded analysis, which included trees and native perennial grasses, suggested that wild plants might maintain higher exudation and rhizodeposition under drought than crop plants, which contrasts with an earlier analysis of only nine studies that suggested the opposite (Preece and Peñuelas, 2016). The variation in direction and magnitude of total C exuded among functional groups is consistent with the emerging view of root exudation as a key functional trait. Specifically, root exudation rate has been proposed to operate along the acquisitive–conservative resource use continuum, based on the ‘fast–slow’ trait-based plant economics spectrum in leaves, stems and roots (Wright *et al.*, 2004; Reich, 2014); but see Oh *et al.* (2024). Several studies have observed that acquisitive (‘fast’) species exude more C than conservative (‘slow’) species and that root exudation rate is strongly related to root morphology (Guyonnet *et al.*, 2018). For instance, root exudation positively correlated with root respiration and N concentration and negatively with root tissue density (Sun *et al.*, 2021). Root exudation also may vary among functional types that harbour different soil microbes, which may be related to the root economics spectrum (Bergmann *et al.*, 2020) and/or the composition of surplus C exuded from roots (Prescott *et al.*, 2020). For example, after 2 years of experimental summer drought, ectomycorrhizal tree species significantly increased root exudation rates early in the growing season and marginally increased later in the growing season, while drought did not significantly affect root exudation in arbuscular mycorrhizal trees (Liese *et al.*, 2018).

### Drought response differed between sampling methods

Drought had a significant positive effect on root total C collected using the exudation trap and soil–hydroponic hybrid methods. The exudation trap method was designed for sampling mature trees in forests (Phillips *et al.*, 2008) and was the only method in our analysis that was used to collect root exudates from trees in the field. The soil–hydroponic hybrid method has often been used in the greenhouse (Ulrich *et al.*, 2022) and allows for growing plants in soil and measuring exudates in a soil-like medium. This method involves excavating roots, applying an exudation trap of glass beads (or another C-free medium) to roots, and collecting exudates after a known period of time. Glass beads do not provide a C source but still provide mechanical pressure on the root system, resembling natural soil conditions. However, disadvantages of this method include concerns that excavation of roots can damage the root system; that transplanting into the C-free medium can result in moisture conditions not represented in the experiment; that the use of a microbial activity inhibitor can affect root exudate composition; findings that glass beads may affect results; and that different root zones and types may release different compound types and amounts (Oburger and Jones, 2018; Döll *et al.*, 2024; Otxandorena-Ieregi *et al.*, 2024). For example, in a recent study certain compounds collected from the apical root region increased in response to stress compared with the subapical root region (Tiziani *et al.*, 2022; Garcia Arredondo *et al.*, 2024). Other methods, such as hydroponic culture-based systems and leachate collection methods, also have drawbacks, including the possibility that the exudates collected originate from plants and microbes so do not accurately reflect plant-only inputs. Additionally, sterile hydroponic environments, nutrient solutions and leaching columns are not representative of the complex plant–microbe–soil interactions that occur in the field. Also, a hydroponic system may contribute to a ‘flushing’ of metabolites that accumulate in response to drought (Canarini *et al.*, 2016). Similarly, the rhizosphere soil suspension method also may not accurately collect plant-derived exudates. Isotope-based tracer and labelling approaches are widely utilized to estimate rhizodeposition, which includes root exudates, dead root cells and mycorrhizal components, but cannot measure root exudation alone or quantify specific chemical compounds. The use of rhizoboxes is another sampling approach that was not included in our dataset (Oburger and Jones, 2018). For example, sorption trap discs are applied directly to roots of plants grown in rhizoboxes, which allow viewing and accessing root systems grown in soil (Bohm *et al.*, 2025). However, the setup is complicated and the recovery efficiency of exudates can be low. Given the distinct advantages and disadvantages associated with different root exudation and rhizodeposition sampling methods, a perfect root exudation sampling method does not exist (Oburger and Jones, 2018; Vives-Peris *et al.*, 2020; Williams *et al.*, 2021). We recommend that studies acknowledge the selected method’s limitations and limit the study’s scope of inference when necessary.

In contrast to the exudation trap and soil–hydroponic hybrid methods, we observed no significant total RR for the isotope-based, leachate collection and soil suspension methods. Given the variation in number of studies, drought intensity ranges and functional types investigated using each method, more research is needed on a broader range of drought intensities and functional types using each collection method. Additionally, root exudation and rhizodeposition best practices as well as systematic comparisons of methodological factors such as duration of exudate collection, type of growth and collection medium, recovery period, root damage extent, growing conditions, sampling volume and presence or absence of microbial activity inhibitors (e.g. antibiotics) are needed and emerging (Williams *et al.*, 2021; McLaughlin *et al.*, 2023, 2025; Otxandorena-Ieregi *et al.*, 2024; Brunn *et al.*, 2025).

### Future directions

Our synthesis revealed knowledge gaps and directions for future research. Despite growing research in this topic, the majority of existing studies focus on grass species and agricultural systems in controlled experimental conditions, indicating the need for more research on trees, shrubs, gymnosperms and wild perennial grasses, and additional research performed in the field. Our results also indicated the need for research at higher drought intensities, either through longer time periods or higher severity, as well as in the field. Long-term field experiments within mixed plant communities that can impose longer droughts across growing seasons are needed to quantify broad-scale drought impacts on plant C allocation, root exudate quantity and quality, and terrestrial C cycling. We also identified the need for more studies to measure not only total C but also compound classes and targeted compounds such as proline, commonly linked to drought response. These studies should also include a broader range of species and ecosystems, across developmental stages, from mixed plant communities, across larger drought intensity ranges and during drought recovery. We encourage future research to take advantage of recent technological advancements and the development of and increasing access to more sensitive metabolomics platforms (e.g. Fourier transform ion cyclotron resonance (FTICR) mass spectrometry; e.g. Ulrich *et al.*, 2022). No sampling method is perfect so its limitations should be acknowledged. Additionally, more studies are needed on combined stressors because combined stress responses can provide non-additive effects which cannot be predicted based on studies of single stressors (Pandey *et al.*, 2015; Tiziani *et al.*, 2022; Choudhary and Senthil-Kumar, 2024). Another factor that could not be investigated in our analysis due to lack of information is that exudates can differ in composition depending on the root zone (Tiziani *et al.*, 2022). Future work should also consider and investigate diurnal and seasonal fluctuations in root-derived C fluxes, the potential threshold soil water content values at which exudation decreases or stops, and concurrent measurements of C assimilation and allocation to improve our understanding of how plants manage and partition C to below-ground processes under drought.

### Conclusions

Our meta-analysis included seven functional types, four ecosystems, five sampling methods and both controlled and field studies, and revealed that total C released as root exudates and rhizodeposition significantly increased in response to drought. This suggests that drought-induced increases in root C can influence the rhizosphere even while C availability in the plant may decline. We also found that drought differentially affects specific compound classes released in root C. Specifically, carbohydrates and organic acids significantly increased in response to drought, suggesting these compound classes may affect plant drought resistance. However, fully understanding root-derived C responses under drought is not possible without further study. This review identified knowledge gaps and future research directions to provide insight into root exudation and rhizodeposition dynamics. Namely, we need research on a greater diversity of functional types across a broader range of drought intensities to identify how drought will affect root C responses, plant–microbe interactions, plant performance, ecosystem functioning and terrestrial C dynamics.

## Supplementary Material

mcag007_Supplementary_Data

## Data Availability

Data will be made available upon request to the corresponding author.

## SUPPLEMENTARY DATA

Supplementary data are available at *Annals of Botany* online and consist of the following. Table S1: 29 publications examining the effect of drought stress on root exudates and rhizodeposition. Publications shaded in grey measured total C in root exudates and rhizodeposition. Table S2: performance of variables in explaining heterogeneity of the 40 total C data points. Literature Cited in the Supplementary Data.

## AI ASSISTANCE

The authors declare no use of AI assistance.
