# Supplementary material for: Drought stress increases total C released from roots
Source: Ann Bot. 2026 Jan 8;137(5):1156–68. doi: 10.1093/aob/mcag007 (PMC13197597; doi:10.1093/aob/mcag007)
Supplement: mcag007_Supplementary_Data [file mcag007_supplementary_data.docx]

**Supplemental Information**

**Table S1**. Twenty-nine publications examined the effect of drought stress on root exudates and rhizodeposition. Publications shaded in gray measured total C in root exudates and rhizodeposition.

|  | **Study** | **Family** | **Genus** | **Species** | **Ecosystem** | **Life History** | **Functional Type** | **Flowering** | **Cotyledon** | **Dev. Stage** | **Cultivated or Wild** | **Exp. Type** | **Rhizodeposit or Root Exudate** | **Method** | **Compound Class** | **Measured** |  |
| --- | --- | --- | --- | --- | --- | --- | --- | --- | --- | --- | --- | --- | --- | --- | --- | --- | --- |
| 1 | (Bakhshandeh *et al.* 2019) | Poaceae | Triticum | *Triticum aestivum* | Agricultural | Annual | Grass | Angiosperm | Monocot | Adult | Cultivated | Controlled | Rhizodeposit | Isotope-based | Organic Carbon | SOC (plant-derived from δ^13^C |  |
| 2 | (Bobille *et al.* 2019) | Fabaceae | Pisum | *Pisum sativum* | Agricultural | Annual | Forb | Angiosperm | Dicot | Adult | Cultivated | Controlled | Rhizodeposit | Soil suspension | Amino Acid | Total amino acids, Proline, Glycine, Valine, Alanine, Isoleucine, Leucine, GABA, Serine, Threonine, Phenylalanine, Aspartate, Glutamate, Asparagine, Glutamine, Homoserine |  |
| 3 | (Brunn *et al.* 2022) | Pinaceae  Fagaceae | Picea  Fagus | *Picea abies*  *Fagus sylvatica* | Forest | Perennial | Broadleaf and Conifer | Angiosperm and Gymnosperm | Dicot and Gymnosperm | Adult | Wild | Field | Exudate | Exudation trap | Organic Carbon | TOC |  |
| 4 | (Calvo *et al.* 2017) | Poaceae | Hordeum | *Hordeum vulgare* | Agricultural | Annual | Grass | Angiosperm | Monocot | Adult | Cultivated | Controlled | Exudate | Leachate Collection | Amino Acid | Total Amino Acids, Proline |  |
|  |  |  |  |  |  |  |  |  |  |  |  |  |  |  | Ion | K^+^ |  |
|  |  |  |  |  |  |  |  |  |  |  |  |  |  |  | Hormone | ABA, Cytokinins, IAA |  |
| 5 | (Calvo *et al.* 2019) | Poaceae | Hordeum | *Hordeum vulgare* | Agricultural | Annual | Grass | Angiosperm | Monocot | Adult | Cultivated | Controlled | Exudate | Leachate Collection | Organic Carbon | DOC |  |
|  |  |  |  |  |  |  |  |  |  |  |  |  |  |  | Carbohydrate | Sucrose, Fructose, Glucose |  |
|  |  |  |  |  |  |  |  |  |  |  |  |  |  |  | Ion | Na^+^ |  |
| 6 | (Canarini and Dijkstra 2015) | Poaceae | Triticum | *Triticum aestivum* | Agricultural | Annual | Grass | Angiosperm | Monocot | Adult | Cultivated | Controlled | Rhizodeposit | Isotope-based | Organic Carbon | ^13^C |  |
| 7 | (Canarini *et al.* 2016) | Asteraceae  Fabaceae | Helianthus  Glycine | *Helianthus annuus*  *Glycine max* | Agricultural | Annual | Forb, Legume | Angiosperm | Dicot | Juvenile | Cultivated | Controlled | Exudate | Soil-hydroponic-hybrid | Organic Carbon | Carbon |  |
|  |  |  |  |  |  |  |  |  |  |  |  |  |  |  | Carbohydrate | Sugars |  |
|  |  |  |  |  |  |  |  |  |  |  |  |  |  |  | Amino Acid | Amino Acids |  |
|  |  |  |  |  |  |  |  |  |  |  |  |  |  |  | Organic Acid | Organic Acid |  |
| 8 | (De Vries *et al.* 2019) | Poaceae  Polygonaceae | Holcus  Rumex | *Holcus lanatus Rumex acetosa* | Grassland | Perennial | Grass, Forb | Angiosperm | Monocot and Dicot | Adult | Wild | Controlled | Exudate | Soil-hydroponic-hybrid | Organic Carbon | TOC |  |
| 9 | (Fuchslueger *et al.* 2014) | Asteraceae  Poaceae  Poaceae  Rosaceae  Fabaceae | Leontodon  Anthoxanthum  Festuca  Alchemilla  Trifolium | *Leontodon hispidus*  *Anthoxanthum odoratum*  *Festuca rubra*  *Alchemilla vulgaris*  *Trifolium repens* | Grassland | Mix | Grass, Forb, Legume | Angiosperm | Mix | Adult | Wild | Field | Rhizodeposit | Isotope-based | Organic Carbon | EOC |  |
| 10 | (Gorissen *et al.* 2004) | Ericaceae | Calluna  Empetrum  Vaccinium | *Calluna vulgaris*  *Empetrum nigrum*  *Vaccinium myrtillus* | Shrubland | Perennial | Shrub | Angiosperm | Dicot | Adult | Wild | Field | Exudate | Isotope-based | Organic Carbon | ^14^C |  |
| 11 | (Henry *et al.* 2007) | Poaceae | Agropyron | *Agropyron cristatum* | Agricultural | Perennial | Grass | Angiosperm | Monocot | Adult | Cultivated | Controlled | Exudate | Leachate Collection | Organic Carbon | TOC |  |
| 12 | (Holz *et al.* 2018) | Poaceae | Zea | *Zea maize* | Agricultural | Annual | Grass | Angiosperm | Monocot | Juvenile | Cultivated | Controlled | Rhizodeposit | Isotope-based | Organic Carbon | ^14^C |  |
| 13 | (Jakoby *et al.* 2020) | Anacardiaceae  Cupressaceae | Pistacia  Cupressus | *Pistacia lentiscus*  *Cupressus sempervirens* | Forest | Perennial | Conifer and Broadleaf Tree | Angiosperm and Gymnosperm | Dicot and Gymnosperm | Adult | Wild | Field | Exudate | Exudation trap | Organic Carbon | TOC |  |
| 14 | (Karlowsky *et al.* 2018) |  | grass  grass  grass  forb  forb | *Deschampsia cespitosa*  *Festuca rubra*  *Dactylis glomerata*  *Leontodon hispidus*  *Geranium sylvaticum* | Grassland | Mix | Grass, Forb, Legume | Angiosperm | Mix | Adult | Wild | Field | Rhizodeposit | Isotope-based | Organic Carbon | EOC |  |
|  |  |  | legume | *Trifolium repens* |  |  |  |  |  |  |  |  |  |  |  |  |  |
| 15 | (Karst *et al.* 2017) | Salicaceae | Populus | *Populus tremuloides* | Forest | Perennial | Broadleaf Tree | Angiosperm | Dicot | Seedling | Wild | Controlled | Exudate | Soil-hydroponic-hybrid | Organic Carbon | TOC |  |
| 16 | (Li *et al.* 2021) | Amaryllidaceae  Poaceae | Allium  Stipa | *Alliyum polyrhizum*  *Stipa krylovii* | Grassland | Perennial | Grass, Forb | Angiosperm | Monocot | Adult | Wild | Field | Exudate | Exudation trap | Organic Carbon | TOC |  |
|  |  |  |  |  |  |  |  |  |  |  |  |  |  |  | Nitrogen Compound | TN |  |
| 17 | (Liang *et al.* 2020) | Poaceae | Oryza | *Oryza sativa* | Agricultural | Annual | Grass | Angiosperm | Monocot | Adult | Cultivated | Field | Exudate | Soil-hydroponic-hybrid | Organic Acid | Total Organic Acids, Succinic Acid, Maleic Acid, Acetic Acid, Citric Acid, Malic Acid, Tartaric Acid, Oxalic Acid, |  |
| 18 | (Liese *et al.* 2018) | Betulaceae  Fagaceae  Fagaceae  Malvaceae  Oleaceae  Rosaceae  Sapindaceae  Sapindaceae | Carpinus  Fagus  Quercus  Tilia  Fraxinus  Prunus  Acer  Acer | *Carpinus betulus*  *Fagus sylvatica*  *Quercus robur*  *Tilia cordata*  *Fraxinus excelsior*  *Prunus avium*  *Acer platanoides*  *Acer pseudoplanatus* | Forest | Perennial | Broadleaf Tree | Angiosperm | Dicot | Juvenile | Wild | Field | Exudate | Exudation trap | Organic Carbon | DOC |  |
| 19 | (Palta and Gregory 1997) | Poaceae | Triticum | *Triticum aestivum* | Agricultural | Annual | Grass | Angiosperm | Monocot | Adult | Cultivated | Controlled | Rhizodeposit | Isotope-based | Organic Carbon | ^13^C |  |
| 20 | (Pereira *et al.* 2021) | Poaceae | Saccharum | *Saccharum officinarum* | Agricultural | Perennial | Grass | Angiosperm | Monocot | Juvenile | Cultivated | Controlled | Exudate | Soil-hydroponic-hybrid | Organic Acid | Citric Acid, Isocitric Acid, Malic Acid |  |
| 21 | (Preece *et al.* 2018) | Fagaceae | Quercus | *Quercus ilex* | Forest | Perennial | Broadleaf and Conifer | Angiosperm and Gymnosperm | Dicot, Gymnosperm | Adult | Wild | Field | Exudate | Exudation trap | Organic Carbon | TOC |  |
| 22 | (Preece *et al.* 2021) | Fagaceae  Pinaceae | Quercus  Pinus | *Quercus ilex*  *Pinus sylvestris* | Forest | Perennial | Broadleaf and Conifer | Angiosperm  Gymnosperm | Dicot  Gymnosperm | Seedling | Wild | Controlled | Exudate | Exudation trap | Organic Carbon | TOC |  |
| 23 | (Sanaullah *et al.* 2012) | Fabaceae  Poaceae  Poaceae | Medicago  Festuca  Lolium | *Medicago sativa*  *Festuca arundinacea*  *Lolium perenne* | Grassland | Perennial | Grass, Legume | Angiosperm | Mix | Juvenile | Wild | Controlled | Rhizodeposit | Isotope-based | Organic Carbon | ^14^C |  |
| 24 | (Schulze and Merbach 2008) | Poaceae | Triticum | *Triticum aestivum* | Agricultural | Annual | Grass | Angiosperm | Monocot | Juvenile | Cultivated | Controlled | Exudate | Isotope-based | Nitrogen Compound | ^15^N |  |
| 25 | (Somasundaram *et al.* 2009) | Poaceae | Zea | *Zea mays* | Agricultural | Annual | Grass | Angiosperm | Monocot | Seedling | Cultivated | Controlled | Rhizodeposit | Isotope-based | Organic Carbon | ^13^C |  |
| 26 | (Svenningsson *et al.* 1990) | Brassicaceae | Brassica | *Brassica napus* | Agricultural | Annual | Forb | Angiosperm | Dicot | Juvenile | Cultivated | Controlled | Exudate | Leachate Collection | Organic Carbon | DOC |  |
|  |  |  |  |  |  |  |  |  |  |  |  |  |  |  | Carbohydrate | Glucose, Fructose, Sucrose, m-inositol |  |
|  |  |  |  |  |  |  |  |  |  |  |  |  |  |  | Amino Acid | Total Amino Acids, Alanine, Glycine, Valine, Serine, Leucine, Isoleucine, Proline, Hydroxyproline, Methionine, Aspartic Acid, Phenylalanine, Ornithine, Glutamic Acid, Lysine, Tyrosine |  |
|  |  |  |  |  |  |  |  |  |  |  |  |  |  |  | Fatty Acid | Total Fatty Acids |  |
|  |  |  |  |  |  |  |  |  |  |  |  |  |  |  | Sterol | Β-sitosterol, Stigmasterol, Campesterol |  |
| 27 | (Ulrich *et al.* 2022) | Poaceae | Bouteloua | *Bouteloua gracilis* | Grassland | Perennial | Grass | Angiosperm | Monocot | Adult | Wild | Controlled | Exudate | Soil-hydroponic-hybrid | Organic Carbon | TOC |  |
|  |  |  |  |  |  |  |  |  |  |  |  |  |  |  |  | Amino Acids |  |
|  |  |  |  |  |  |  |  |  |  |  |  |  |  |  |  |  |  |
|  |  |  |  |  |  |  |  |  |  |  |  |  |  |  |  | Organic Acids |  |
|  |  |  |  |  |  |  |  |  |  |  |  |  |  |  |  | Sugars |  |
|  |  |  |  |  |  |  |  |  |  |  |  |  |  |  |  | Metabolites |  |
| 28 | (Xia *et al.* 2020) | Salicaceae | Populus | *Populus cathayana* | Forest | Perennial | Broadleaf Tree | Angiosperm | Dicot | Juvenile | Wild | Controlled | Exudate | Soil Suspension | Organic Acid | Citrate |  |
| 29 | (Zhu and Cheng 2013) | Asteraceae  Fabaceae | Helianthus  Glycine | *Helianthus annuus*  *Glycine max* | Agricultural | Annual | Legume | Angiosperm | Dicot | Adult | Cultivated | Controlled | Rhizodeposit | Isotope-based | Organic Carbon | ^13^C |  |
|  |  |  |  |  |  |  |  |  |  |  |  |  |  |  |  |  |  |

**Table S2.** Performance of variables in explaining heterogeneity of the 40 total C data points. The first row contains the original random-effects model with no variables. The variables that explained the most variance were ecosystem, flowering type, cotyledon type, drought intensity, and functional type (i.e., resulted in the smallest residual **τ**^2^ and largest % reduction in **τ**^2^). Qtotal is the total heterogeneity between RRs and equals the sum of Qwithin and Qbetween (Qtotal = Qwithin + Qbetween). Qwithin is **the** heterogeneity **within subgroups**. Qbetween is **the variance explained by the variable between subgroups. τ**^2^ refers to the pooled variance that remains within subgroups after grouping by each variable (i.e., a low **τ**^2^ indicates that that variable explains nearly all of the variance or that points within subgroups are homogeneous). **P-values <0.05 are bolded and** used to indicate that subgroups differed significantly and that the variable is statistically important in explaining variance. Variables (categories) included: ecosystem (agricultural, grassland, shrubland, forest), life history (annual, perennial), flowering type (angiosperm, gymnosperm), cotyledon type (monocot, dicot), functional type (grass, forb, legume, grass/forb/legume mix, broadleaf tree, conifer, shrub), developmental stage (seedling, juvenile, adult), species cultivated for agriculture or not (cultivated, wild), experimental setup (greenhouse, field), root C type (rhizodeposition, root exudate), and sampling method (exudation trap, soil-hydroponic-hybrid, isotope-based, leachate collection, soil suspension).

| **Variable** | **# of subgroups** | **Qtotal** | **Qwithin** | **Qbetween** | **τ²** | **% reduction in τ²** | **p-value** |
| --- | --- | --- | --- | --- | --- | --- | --- |
| **Original model (no variables)** | -- | 62.37 | -- | -- | 0.1681 | -- | -- |
| **Ecosystem** | 4 | 99.90 | 67.45 | 32.45 | 0.0807 | 51.99 | **<0.001** |
| **Flowering type** | 2 | 101.66 | 60.36 | 41.29 | 0.0785 | 53.3 | **<0.001** |
| **Cotyledon type** | 4 | 88.99 | 67.88 | 21.11 | 0.0970 | 42.30 | **0.0001** |
| **Drought intensity** | -- | 87.13 | 56.46 | 30.67 | 0.1003 | 40.33 | **<0.001** |
| **Functional type** | 6 | 70.57 | 43.33 | 27.24 | 0.1392 | 17.19 | **0.0001** |
| **Life history** | 3 | 59.14 | 54.14 | 4.99 | 0.1822 | -8.39 | 0.0824 |
| Root C type | 2 | 58.52 | 55.98 | 2.54 | 0.1851 | -10.11 | 0.1107 |
| **Developmental stage** | 3 | 62.11 | 61.78 | 0.33 | 0.1691 | -0.59 | 0.8467 |
| **Cultivation status** | 2 | 58.40 | 54.08 | 4.32 | 0.1856 | -10.41 | **0.0376** |
| **Experimental setup** | 2 | 57.82 | 56.44 | 1.38 | 0.1885 | -12.14 | 0.2395 |
| **Sampling method** | 4 | 57.46 | 49.37 | 8.09 | 0.1902 | -13.15 | **0.0441** |

**Literature cited**

**Bakhshandeh S, Corneo PE, Yin L, Dijkstra FA**. **2019**. Drought and heat stress reduce yield and alter carbon rhizodeposition of different wheat genotypes. *Journal of Agronomy and Crop Science* **205**: 157–167.

**Bobille H, Fustec J, Robins RJ, Cukier C, Limami AM**. **2019**. Effect of water availability on changes in root amino acids and associated rhizosphere on root exudation of amino acids in Pisum sativum L. *Phytochemistry* **161**: 75–85.

**Brunn M, Hafner BD, Zwetsloot MJ, *et al.*** **2022**. Carbon allocation to root exudates is maintained in mature temperate tree species under drought. *New Phytologist* **235**: 965–977.

**Calvo OC, Franzaring J, Schmid I, Fangmeier A**. **2019**. Root exudation of carbohydrates and cations from barley in response to drought and elevated CO 2. *Plant and Soil* **438**: 127–142.

**Calvo OC, Franzaring J, Schmid I, Müller M, Brohon N, Fangmeier A**. **2017**. Atmospheric CO2 enrichment and drought stress modify root exudation of barley. *Global change biology* **23**: 1292–1304.

**Canarini A, Dijkstra FA**. **2015**. Dry-rewetting cycles regulate wheat carbon rhizodeposition, stabilization and nitrogen cycling. *Soil Biology and Biochemistry* **81**: 195–203.

**Canarini A, Merchant A, Dijkstra FA**. **2016**. Drought effects on Helianthus annuus and Glycine max metabolites: from phloem to root exudates. *Rhizosphere* **2**.

**De Vries FT, Williams A, Stringer F, *et al.*** **2019**. Changes in root‐exudate‐induced respiration reveal a novel mechanism through which drought affects ecosystem carbon cycling. *New Phytologist* **224**: 132–145.

**Fuchslueger L, Bahn M, Fritz K, Hasibeder R, Richter A**. **2014**. Experimental drought reduces the transfer of recently fixed plant carbon to soil microbes and alters the bacterial community composition in a mountain meadow. *New Phytologist* **201**: 916–927.

**Gorissen A, Tietema A, Joosten NN, *et al.*** **2004**. Climate change affects carbon allocation to the soil in shrublands. *Ecosystems* **7**: 650–661.

**Henry A, Doucette W, Norton J, Bugbee B**. **2007**. Changes in crested wheatgrass root exudation caused by flood, drought, and nutrient stress. *Journal of environmental quality* **36**: 904–912.

**Holz M, Zarebanadkouki M, Kaestner A, Kuzyakov Y, Carminati A**. **2018**. Rhizodeposition under drought is controlled by root growth rate and rhizosphere water content. *Plant and Soil* **423**: 429–442.

**Jakoby G, Rog I, Megidish S, Klein T**. **2020**. Enhanced root exudation of mature broadleaf and conifer trees in a Mediterranean forest during the dry season. *Tree Physiology* **40**: 1595–1605.

**Karlowsky S, Augusti A, Ingrisch J, *et al.*** **2018**. Land use in mountain grasslands alters drought response and recovery of carbon allocation and plant‐microbial interactions. *Journal of Ecology* **106**: 1230–1243.

**Karst J, Gaster J, Wiley E, Landhäusser SM**. **2017**. Stress differentially causes roots of tree seedlings to exude carbon. *Tree Physiology* **37**: 154–164.

**Li C, Liu L, Zheng L, *et al.*** **2021**. Greater soil water and nitrogen availability increase C: N ratios of root exudates in a temperate steppe. *Soil Biology and Biochemistry* **161**: 108384.

**Liang S, Wang Y, Zhang H, Yun X, Wu Y**. **2020**. Response of root-exuded organic acids in irrigated rice to different water management practices. *Eurasian Soil Science* **53**: 1572–1578.

**Liese R, Lübbe T, Albers NW, Meier IC**. **2018**. The mycorrhizal type governs root exudation and nitrogen uptake of temperate tree species. *Tree Physiology* **38**: 83–95.

**Palta J, Gregory P**. **1997**. Drought affects the fluxes of carbon to roots and soil in 13C pulse-labelled plants of wheat. *Soil Biology and Biochemistry* **29**: 1395–1403.

**Pereira LB, de Oliveira Gambarini VM, de Menezes AB, Ottoboni LM, Vicentini R**. **2021**. Responses of the sugarcane rhizosphere microbiota to different levels of water stress. *Applied Soil Ecology* **159**: 103817.

**Preece C, Farré-Armengol G, Llusià J, Peñuelas J**. **2018**. Thirsty tree roots exude more carbon. *Tree Physiology* **38**: 690–695.

**Preece C, Farre-Armengol G, Verbruggen E, Penuelas J**. **2021**. Interactive effects of soil water content and nutrients on root exudation in two Mediterranean tree species. *Soil Biology and Biochemistry* **163**: 108453.

**Sanaullah M, Chabbi A, Rumpel C, Kuzyakov Y**. **2012**. Carbon allocation in grassland communities under drought stress followed by 14C pulse labeling. *Soil Biology and Biochemistry* **55**: 132–139.

**Schulze J, Merbach W**. **2008**. Nitrogen rhizodeposition of young wheat plants under elevated CO 2 and drought stress. *Biology and Fertility of Soils* **44**: 417–423.

**Somasundaram S, P. Rao T, Tatsumi J, Iijima M**. **2009**. Rhizodeposition of Mucilage, Root Border Cells, Carbon and Water under Combined Soil Physical Stresses in Zea mays L. *Plant Production Science* **12**: 443–448.

**Svenningsson H, Sundin P, Liljenberg C**. **1990**. Lipids, carbohydrates and amino acids exuded from the axenic roots of rape seedlings exposed to water‐deficit stress. *Plant, Cell & Environment* **13**: 155–162.

**Ulrich DEM, Clendinen CS, Alongi F, *et al.*** **2022**. Root exudate composition reflects drought severity gradient in blue grama (Bouteloua gracilis). *Scientific Reports* **12**: 12581.

**Xia Z, He Y, Zhou B, Korpelainen H, Li C**. **2020**. Sex-related responses in rhizosphere processes of dioecious *Populus cathayana* exposed to drought and low phosphorus stress. *Environmental and Experimental Botany* **175**: 104049.

**Zhu B, Cheng W**. **2013**. Impacts of drying–wetting cycles on rhizosphere respiration and soil organic matter decomposition. *Soil Biology and Biochemistry* **63**: 89–96.
